# Long-Delayed Amended Dental Bill

**Published:** 1899-02

**Authors:** 


The Victorian Dental Board recently interviewed the Chief Secretary on the subject of the long-delayed Amended Dental Bill, and secured a promise that a measure would be ntroduced this session in time to be finally disposed of. The deputation expressed the strong objection to a proposed clause, prohibiting any member of the Board from being an officer, lecturer, examiner, or member of the Council or Committee of any dental college or hospital, on the ground that many of the best known and most experienced dentists of Melbourne were connected in one or other of those capacities with a dental college or hospital, and the clause would mean their withdrawal from either the Board or the institution, with consequent loss and injury to one or the other. The Chief Secretary promised to place these representations under the consideration of the Cabinet.—*Pharmaceutical Journal.*

